# Equilibrium and Kinetic Study of Lead and Copper Ion Adsorption on Chitosan-Grafted-Polyacrylic Acid Synthesized by Surface Initiated Atomic Transfer Polymerization

**DOI:** 10.3390/molecules23092218

**Published:** 2018-09-01

**Authors:** Carlos David Grande-Tovar, William Vallejo, Fabio Zuluaga

**Affiliations:** 1 Grupo de Fotoquímica y Fotobiología, Universidad del Atlántico, Puerto Colombia 81007, Colombia; carlosgrande@mail.uniatlantico.edu.co; 2 Departamento de Química, Universidad del Valle, Santiago de Cali 760032, Colombia; hector.zuluaga@correounivalle.edu.co

**Keywords:** ATRP, chitosan grafted polyacrylic acid, heavy metals, adsorption, water treatment

## Abstract

In this work, we synthesized chitosan grafted-polyacrylic acid (CS-*g*-PA) through surface-initiated atom transfer radical polymerization (SI-ATRP). We also studied the adsorption process of copper and lead ions onto the CS-*g*-PA surface. Adsorption equilibrium studies indicated that pH 4.0 was the best pH for the adsorption process and the maximum adsorption capacity over CS-*g*-PA for Pb^2+^ ions was 98 mg·g^−1^ and for Cu^2+^ it was 164 mg·g^−1^, while for chitosan alone (CS), the Pb^2+^ adsorption capacity was only 14.8 mg·g^−1^ and for Cu^2+^ it was 140 mg·g^−1^. Furthermore, the adsorption studies indicated that Langmuir model describes all the experimental data and besides, pseudo-second-order model was suitable to describe kinetic results for the adsorption process, demonstrating a larger kinetic constant of the process was larger for Pb^2+^ than Cu^2+^. Compared to other adsorbents reported, CS-*g*-PA had comparable or even superior adsorbent capacity and besides, all these results suggest that the new CS-*g*-PA polymers had potential as an adsorbent for hazardous and toxic metal ions produced by different industries.

## 1. Introduction

High content of metals (e.g., lead, cadmium, chromium, copper, nickel, arsenic, etc.) in water is dangerous to human health; currently, environmental research is directed to develop efficient methods for metal removal without causing additional subsequent contamination problems and besides, an important environmental concern [1]. Primarily, both lead and copper are reported among the most toxic metals to the environment; these metals have drawn much attention owing to its impact on the public health and toxicity, and for that reason, the United States Environmental Protection Agency (EPA), has classified some heavy metal ions as priority pollutants due to their high degree of toxicity and hazard when they are present in water and recently has published “Use of Lead Free Pipes, Fittings, Fixtures, Solder and Flux for Drinking Water” [2]. The main sources of lead and copper are wastewater discharge, mining activities, phosphate fertilizers, the textile and leather industries, plumbing manufacturing, electric wires, pigments, electroplating, municipal waste, sewage waste, welding, and galvanized steel, among others [3]. Lead, for example, represents a serious hazard because of its accumulation capacity and ability to damage the central nervous system. This metal, commonly found in minerals such as galena and cerussite, is used for the manufacture of batteries, welding, paints, pyrotechnics, pottery, glassware and mining. According to the WHO, the maximum level of lead in blood should be 0.015 mg/L [4]. Copper is a metal used in electrical, electronics and metal materials, due to its high electrical conductivity, ductility and malleability. It is also necessary for some biological processes (i.e., myelin formation of hemoglobin and excessive blood clotting prevention acting as a coenzyme of the immune system and regulating the production of melanin [5]), however, high concentrations of this element in the human body can affect health, causing headaches, vomiting, liver, blood, vessels and kidney damage; furthermore, exposure to copper for a long time can irritate the nose, mouth, eyes and respiratory system; the permitted level given by the WHO for Cu (II) into drinking water is 1.3 mg/L [6,7]. Different approaches have been employed to solve the growing need for lead and copper ion removal from water, including chemical precipitation and coagulation, membrane separation, ion exchange, and adsorption [7,8]. These methods each have distinct limitations and disadvantages when heavy metals are removed from groundwater. However, during the last years, adsorption processes have been shown to be effective and economical treatment processes, making them the most promising methodology. Table 1 lists some properties of the main technologies applied to metal removal [9].

Generally, materials that are able to remove lead and copper ions bear nitrogen, oxygen, and sulfur functional groups as the binding sites. Therefore, natural adsorbents, agricultural waste, and biopolymers have attracted considerable attention to remove heavy metal ions from wastewater due to their low cost and abundance in Nature and high efficiency [17]. Chitosan is a biopolymer of high interest because of its biocompatibility and antimicrobial effects against different microorganisms, currently obtained from the partial deacetylation of chitin, a polysaccharide obtained from the exoskeleton of crustaceans and the mycelium of some fungi [18]. Chitosan can form films and has several cross-linking points, such as nitrogen atoms, that also have chelating properties, and for this reason it has been used in different preparations (beads, nanobeads, nanoparticles, etc.) to remove heavy metal from different sources [19]. Chitosan beads, for example, have been used to remove effectively lead and copper [20,21,22]. Wan and Fatinathan have reported removal efficiencies greater than 60% for Pb(II) and Cu(II) by using chitosan beads, chitosan—GLA and chitosan-alginate beads [15,16]; Laus and Fávare reported removal of Cu(II) and Cd(II) by using chitosan crosslinked with epichlorohydrin–triphosphate; they reported % removal between (50–90%) for Cu(II) [23] and besides, Futalan et al. studied competitive adsorption of Cu(II), Pb(II), and Ni(II) by using chitosan immobilized on bentonite; they reported 80% to Pb(II) and 50% to Cu(II) removal [24]. All these reports indicate that chitosan has good adsorption capability, however, due to its weak mechanical properties, chemical and physical modifications must be carried out to improve its physicochemical and mechanical properties [16].

Surface-initiated polymerization has emerged as one of the most important techniques to modify the surface properties of materials, with important applications in the fields of optoelectronics, biosciences, and engineering [25]. Surface-initiated atomic radical polymerization, SI-ATRP, is a versatile method for the preparation of different hybrid materials, with a particular control over molecular weight and polydispersity, allowing control over molecular architectures [26]. SI-ATRP does not require stringent experimental conditions like other ionic living polymerization techniques having a high versatility for the polymerization of different monomers. Polymethacrylic acid brushes, for example, have been prepared by SI-ATRP using a two step procedure with protective groups to avoid catalyst poisoning [27,28]. However, the use of protecting groups in some cases could be more expensive and tiresome process. Water treatment with surface modified adsorbents such as polyacrylic acid could be interesting to improve the adsorption properties, resulting from the insolubility, higher adsorption capacities and ease of the purification process (a simple filtration at the end) to remove pollutants.

In this work, we report the surface modification of chitosan by surface-initiated ATRP with poly- (sodium acrylate), in order to obtain directly a chitosan-based material with an increased ability to remove heavy metals such as lead and copper, without the need of extra steps.

## 2. Results and Discussion

### 2.1. Synthesis

Direct ATRP polymerization of acid monomers can be affected by the reaction of the monomer with metal ions to form complexes that are catalytically ineffective [29]. Several authors have synthesized polymer brushes such as polymethacrylic acid and polyacrylic acid using *tert*-butyl acrylate and *tert*-butyl methacrylate via ATRP, using pyrolysis or hydrolysis of the *tert*-butyl ester protective group [30]. However, this methodology introduces additional steps and results in the loss of some polymer chains from the surface; it is clear that direct insertion of the acidic polymer chains by ATRP is a challenge. One possibility is to use a previously reported procedure that uses sodium salts of the acids and later acidification [29]. The SI-ATRP modification of chitosan particles was previously published by our group, but it is briefly discussed here. As we published earlier, the polymerization reaction of sodium acrylate was first conducted on silicon wafer surfaces modified by spin-coated chitosan. The polymerization conditions were: 100:2:0.4:5:1400 for NaAA:CuBr:CuBr_2_:PMDETA in a mixture of water/methanol (1:1). However, with an increasing of the molar ratio to 300:2:0.4:1400 for NaAA:CuBr:CuBr_2_:PMDETA and a decrease in the temperature of the system from 60 °C to 25 °C, the thickness of the system is increased as a result of a decreasing of the hydrolysis chain from the silicon surfaces. Details of characterization are reported in previous work by Grande et al. [31].

### 2.2. Chitosan Characterization

#### 2.2.1. FT-IR Characterization

**Figure 1:**
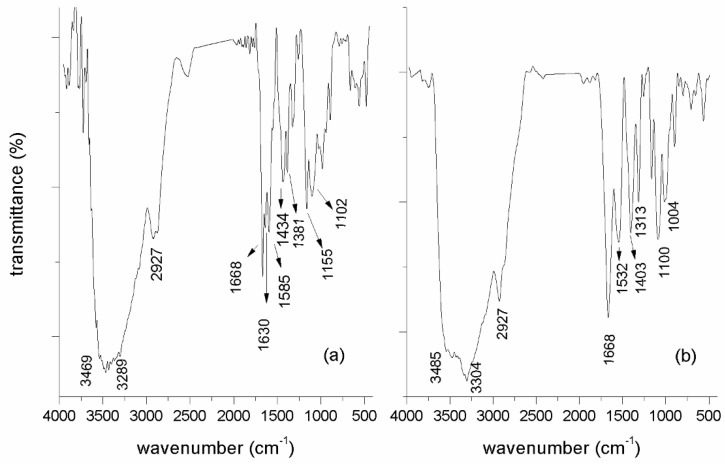
FTIR spectrum in KBr of (**a**) CS-*g*-BIB, and (**b**) CT-*g*-PA.

Surface modification of CS with 2-BIB was probed by FTIR (Figure 1a). The spectrum of the modified chitosan in solution shows the characteristic band at 1667 cm^−1^ of the stretching vibration of the C=O groups of amides, after functionalizing the chitosan, which overlaps with the N−H deformation. At 3400 cm^−1^ a wide strong band is observed, due to the stretch of the −OH typical of alcohols that overlaps the typical N−H stretch of amides. Between 2927 and 2864 cm^−1^ the stretching is observed due to the aliphatic tension vibration −CH; at 1719 cm^−1^, a band due to the stretching vibration of the C=O moieties of esters is observed, which confirms the reaction of the −OH groups of the chitosan with the ATRP initiator. Between 1620 and 1590 cm^−1^, two strong bands characteristic of primary amides are observed due to the deformation of the N−H bonds; between 1442 and 1323 cm^−1^, the deformation vibration of −O−H is apparent; between 1158 and 1100 cm^−1^, the typical CO stretch of esters can be observed, with bands of moderate intensity that could be overlapped with the stretching vibration of the CN bond, typical of amines (due to the amino groups that did not react with the ATRP initiator) [32]. In Figure 1b, two fundamental facts attributable to the polymerization of acrylic acid can be observed. Firstly, the widening of the band due to the vibration of the −OH and N−H bonds of amides, indicating the presence of more −OH groups typical of carboxylic acids. On the other hand, the presence of more carbon atoms on the surface of the chitosan from PA grafted causes an increase in the band due to the stretching of aliphatic −CH, present in the acrylic acid. A new wide band present at 1545 cm^−1^ is due to carboxylic acid anions (which is the state of the pH of the polymer reaction) as a consequence of the immobilization of PA on the surface of chitosan. Between 1083 and 1008 cm^−1^, two bands can be seen that can be assigned to the C-O stretching vibrations of the carboxylic acids, suggesting the effective immobilization of the PA through the ATRP polymerization reaction. The band from the primary amino groups of amines at 1158 cm^−1^ is reduced, which is attributed to the effective formation of the graft copolymer on the surface of the chitosan. These results suggest that the formation of modified chitosan with poly (acrylic acid) was carried out successfully, as observed in Figure 1 [31].

#### 2.2.2. XPS Characterization

**Figure 2:**
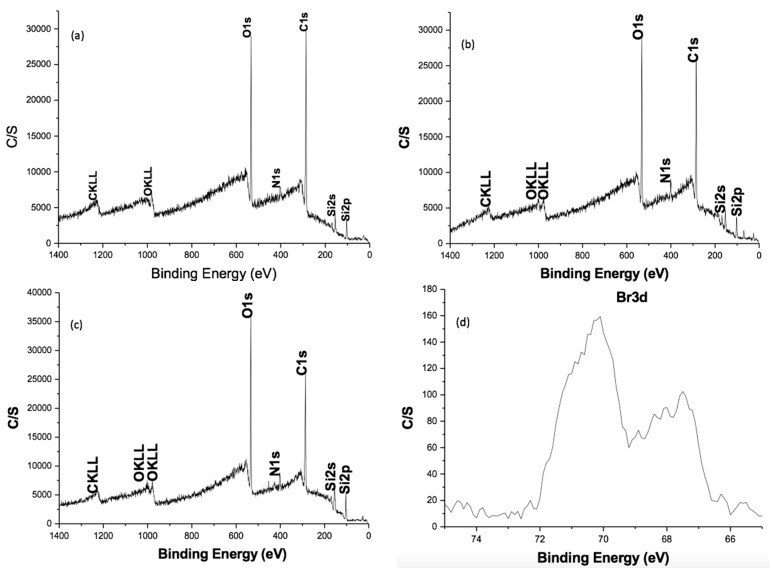
Wide scan photoelectron spectrum of the (**a**) CS, (**b**) CS-BIB initiator (**c**) CS-*g*-PA and (**d**) high resolution spectrum of the Br in the CS-BIB initiator.

The XPS technique was also used to characterize the surface modification process via ATRP with polyacrylic acid. In this case, a solution of chitosan (1 mg/mL) was deposited onto a silica wafer surface using spin-coating technique and evaporated by rotating at 2300 rpm for 30 s. The resulting silica surface was used to carry out the modification process with the ATRP initiator, as described in the experimental part, and subsequently, the polymerization was conducted similarly for the chitosan particles. Figure 2 shows a typical wide-scan spectrum. Typical binding energy peaks for chitosan at 286.1 eV for C1s (69.13%), 534 eV for O 1s (24.66%), and 399 eV for N 1s (4.96%) (Figure 2a), the peak at 70 eV for Br 3d (1.25%) is observed after 2-BIB reaction, confirming the immobilization with the ATRP initiator (Figure 2b), high-resolution analysis of peak Br 3d is shown in Figure 2d [33,34]. However, after PA polymerization, the peak for bromine is absent, accompanied of an increment in the peak, due to O1s increased up to 33.89%, a consequence of the immobilization of poly (acrylic acid), while that the peak of C1s decreased to 56.26% (Figure 2c). The predicted percentage composition of a surface modified with chitosan would show that for carbon, would be around 54.53% and for oxygen 36.32%, very close to the values obtained for CS-*g*-PA, while it differs considerably from pristine CS. The absence of Br 3d in xps analysis (Figure 2c) still in the high-resolution analysis, shows that polymerization was carried out effectively [30]. Moreover, in an attempt to confirm the above, the CS was modified with PMMA, using the same procedure that for PA, and the surface was analyzed by XPS. Composition gave for the C1s 66.07%, N1s 4.33%, and for O1s 29.60%, very close to the predictions of a surface modified with PMMA that would give for carbon 58.80%, and 31.33% for oxygen, theoretically. These results agree with the findings by IR spectroscopy, confirming the immobilization of the polymer on the surface of the chitosan powder.

### 2.3. Initial pH Effect on Adsorption Process

**Figure 3:**
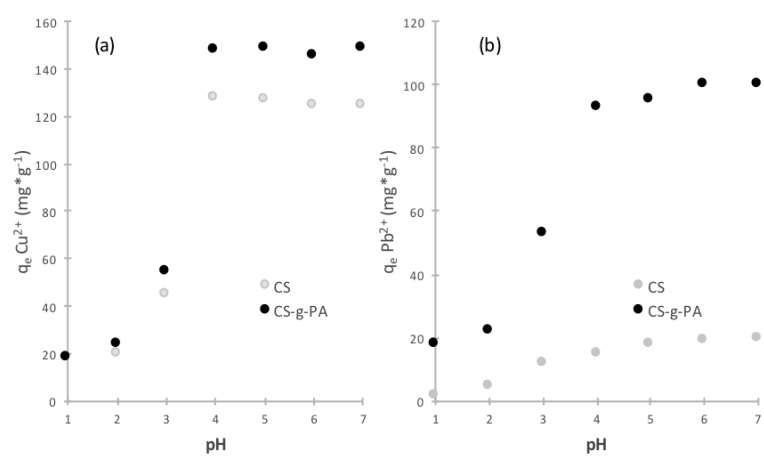
The Effect of initial pH on the adsorption of (**a**) Cu^2+^ and (**b**) Pb^2+^ on CS and CS-*g*-PA (adsorption time: 24 h; Initial amount of adsorbent: 25 mg, initial volume of samples: 50 mL, at 25 °C).

The amount of ions adsorbed on adsorbent’s surface is normally described by the following mass balance equation:

(1) $$q_{t_{i}\;}=\frac{\sum_{i=1}^{n}\left(C_{t_{i-1}}-C_{t_{i}}\right)V_{t_{i-1}}}{m}\;$$

where $C_{t_{i-1}}\;$ is the initial concentration of the ion, $C_{t_{i}\;}$ is the concentration of the ion at time *t*, $V_{t_{i-1}}\;$ is the initial volume of the solution and *m* is the mass of the adsorbent. We studied the adsorption capacity (Figure 3) as a function of pH of lead and copper using chitosan (CS) and chitosan grafted-poly acrylic acid (CS-*g*-PA) prepared by SI-ATRP. Figure 3 illustrates two important aspects: first, an increase in the initial pH of the solution increases the maximum adsorption capacity for both, CS and CS-*g*-PA; however, adsorption capacity is much higher CS-*g*-PA than for CS. From pH 1 to 2, CS-*g*-PA had a slightly greater adsorption, possibly because the chitosan is unstable and tends to solubilize and the acrylate groups are protonated, exhibiting inefficiency to form complexes with ions. However, with the increase of the pH from 4.0–7.0, a dramatic increase occurs in the adsorption capacity of copper ions of CS and CS-*g*-PA, from 45.72 to 127.65 and 53.06 mg/g to 147.65 mg/g, while for the lead, an increase from 10.56 to 13.24 and 53.06 to 93.04 occurred. Above pH 4.0 it becomes apparent that more carboxylate groups are ionized resulting in an increase in the affinity for cations by electrostatic interactions [35].

Different reports have suggested that the mechanism for lead and copper complexation by chitosan/PVA and chitosan/cellulose hydrogels could be the result of a combination of chemical complexation, ion-exchange, and non-specific electrostatic interaction, depending on the solution pH [36]. Furthermore, several authors have reported that chitosan and chitosan beads have positive z potentials in acidic solutions and negative z potentials in basic solutions, with a point of zero z potential at about pH 6.6 close to the pKa values of 6.3–6.7 for the amino group in chitosan reported [37,38]. Hence, the z potentials of CS and CS-*g*-PA result from the protonation/deprotonation of the amine groups on the chitosan [39]. For the other side, it may be expected that lead and copper ion adsorptions on both CS and CS-*g*-PA can be enhanced with the increase of solution pH values, but CS-*g*-PA would show better adsorption performance for lead and copper ions than CS, due to the more attractive electrostatic interactions between CS-*g*-PA and the metal ions [40].

### 2.4. Adsorption Isotherms

Adsorption isotherms describe how the species in solution interact with the adsorbent and also the maximum sorption capacity of the chemical species in solution. Different sorption models are used to fit the experimental data. In this study, Langmuir, Freundlich and Temkin isotherms were employed to describe the lead and copper adsorption equilibrium. In the Langmuir isotherm model, the adsorption process is considered that the adsorbent surface can be covered uniformly with a monolayer of adsorbate; this model assumes a surface with homogeneous binding sites, equivalent sorption energies, and no interactions between adsorbed species; the isotherm is expressed by Equation (2) [41]:

(2) $$\;q_{e}=\frac{q_{m}K_{L}C_{e}\;}{1+K_{L}C_{e}}\;$$

where *C_e_* is the equilibrium concentration of the adsorbate, *q_e_* is the equilibrium amount of adsorbate per gram of absorbent and *q_m_* is the maximum amount of adsorbate per gram of absorbent; *K_L_* is the Langmuir constant (represents kinetic coefficients of adsorption and desorption); *K_L_* is related to the adsorption rate. On the other hand, Freundlich isotherm is an empirical model that describes the process as a multilayer absorption, where the isotherm is expressed by Equation (3):

(3)  qe=kFCe1n  

where *k_F_* is the Freundlich constant (an indicator of adsorption capacity, the greater the maximum capacity, the greater is *k* value); furthermore, the ratio 1/*n* is a measure of the intensity of the adsorption [42]. The *n* and *k_F_* values are empirical constants and they are specific to the system adsorbent/adsorbate.

The Temkin isotherm fits the follow mathematic expression:

(4) $$\;q_{e}=\frac{RT\;}{b}\ln(AC_{e})\;$$

where *C_e_* is the equilibrium concentration of the adsorbate, *q_e_* is the equilibrium amount of adsorbate per gram of absorbent; *A* is the Temkin isotherm constant and *b* is the Temkin related to the heat of adsorption; *T* is the absolute temperature, *R* is the gas constant. The Temkin isotherm assumes that the heat of adsorption of all molecules would decrease linearly rather than logarithmic with coverage by ignoring extremely low and very high values of concentration and besides, it assumes a uniform distribution of bounding energy up to some maximum bonding energy [43,44]. 

**Figure 4:**
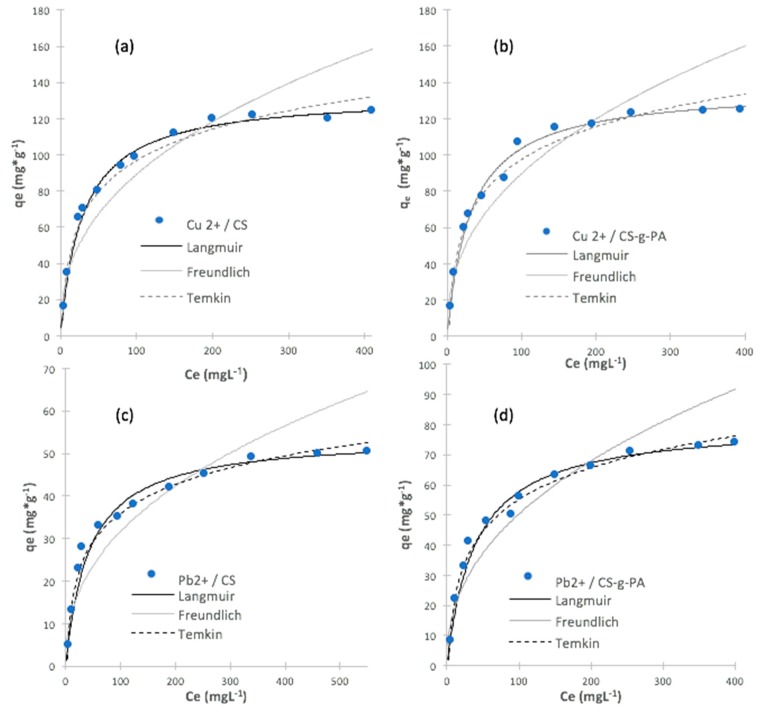
Non-linear theoretical model fitting for copper adsorption on (**a**) CS; (**b**) CS-*g*-TA; and theoretical model fitting for lead adsorption on (**c**) CS and (**d**) CS-*g*-TA. (pH: 4.0; adsorption time: 24 h; initial adsorbent quantity: 25 mg; initial volume: 50 mL; temperature: 25 °C).

Figure 4 shows the sorption equilibrium of lead and copper ions on CS and CS-*g*-PA surfaces. Fitting results are shown for the three theoretical adsorption models, and Table 2 lists the fitting parameters for the three models (information about the Freundlich and Temkin models can be found in the Supporting Information).

Although the correlation coefficient (R^2^) is commonly used for determination of the best isotherm fit, this indicator is limited to solving isotherm models that present linear plots. We used the average relative error (ARE) function to determine the isotherm model most suitable for representing the experimental data; this error function attempts to minimize the fractional error distribution across the entire concentration range, ARE function is described by [45,46,47]:

(5) $$\;ARE=\frac{100\;}{n}\overset{n}{\underset{i=1}{\sum }}\frac{\left|q_{e,cal}-q_{e,meas}\right|}{q_{e,meas}}\;$$

where, *q_e,cal_* is the calculated value, *q_e,meas_* is measured value and n is the number of data points. This model to error analyses were applied by other authors in order to verify which model presented better isotherm adjustment for chitosan and rice husk ash dye adsorptions [46,47]. Figure 4 and Table 2 show that for both metal ions, the Langmuir model fits the experimental results better the than the Freundlich and Temkin models, (largest R^2^ and fewer ARE values); furthermore, Figure 4 shows that the maximum Pb^2+^ adsorption capacity (*q_m_*) increased from *q_m_* = 54 mg/g to 81 mg/g after the grafting process. For Cu^2+^, adsorption capacity (*q_m_*) showed a little improvement from *q_m_* = 133 mg/g to 136 mg/g, after the grafting process. Our results indicate the grafting process improves the maximum capacity for Pb^2+^ and Cu^2+^ ions (additional information can be found in supporting information).

These results indicate that the surface of the adsorbent is quite uniform with many equivalent sorption sites, as a result of the grafting process. It is possible that the sorption process occurs through the same mechanism with a monolayer formed through the surface of adsorbate without deposited molecules upon others already adsorbed. For both metal, adsorption increased proportionally with concentration, suggesting that the active sites of the adsorbent where available for the ions present in the solution, but after a concentration of 200 ppm (for copper) and 300 ppm (for lead), the adsorption value becomes constant, because of the saturation of the active sites of the adsorbent. The adsorption on CS-*g*-PA surface in both cases was higher than CS, indicating that acrylate grafting on chitosan surface improved ion adsorption capacity as compare to chitosan without modifications. From the results, it is possible to observe that in the adsorption of Pb^2+^ and Cu^2+^, the adsorption capacity of CS-*g*-PA is higher compared to CS. However, for the Cu^2+^ species, the effect could be much more limited in the CS-*g*-PA compared with the unmodified CS, because the nitrogens of the −NH_2_ groups, could have a greater affinity for the SO_4_^2−^ groups, a greater charge and bond compensation, generating complexes between several copper ions and a greater capacity of aggregation, compared with the modified chitosan, which does not have much nitrogen available after the modification. On the other hand, carboxylate groups at higher pH do not have too much affinity compared to the previous case. Another possible explanation, could be that in the case of Pb^2+^ the compound that forms at high pH, which is Pb(OH)_2_, is insoluble at pH higher than 8, while in the case of copper, the compound Cu(OH)_2_ begins becomes very insoluble at a pH greater than 6, affecting its adsorption capacity because an increased repulsion with the carboxylate groups [48,49].

Table 1 shows that some adsorbents, such as activated carbon and chitosan, have been used for the removal of Pb(II) and Cu(II) with removal efficiencies of up to 85%, at pH 6.0 and 4.5, respectively, exhibiting an economic, fast and efficient method for the recovery of metals, after the treatment [14,15,16]. On the other hand, different sources from biomass (i.e., wheat bran) have been used to removal of Cu(II), with a removal *q_m_* ranging from 17.4 mg·g^−1^ to 51.5 mg·g^−1^, depending whether they are dehydrated or not [50,51]. Fruit peels such as orange (*q_m_* = 50.94 mg·g^−1^; [52]) and mango (*q_m_* = 46.09 mg·g^−1^; [53]) and tea tree residues (*q_m_* = 8.64 mg·g^−1^; [54]), biomass sources from microorganisms have also been used such as: (a) the bacterium *Pseudomonas putida* (*q_m_* = 89.60 mg·g^−1^; [55]); (b) *Streptomyces coelicolor* (*q_m_* = 66.7 mg·g^−1^; [56]); (c) fungi as *Aspergillus niger* (*q_m_* = 26 mg·g^−1^; [57]); (d) algae as *Sargassum* sp. (*q_m_* = 87.1 mg·g^−1^; [58]) and *Spyrogyra* (*q_m_* = 133.3 mg·g^−1^, [59]). In our study, for CS the *q_m_* = 133 mg·g^−1^ and for the CS-*g*-PA it was *q_m_* = 136 mg·g^−1^, respectively, demonstrating that CS and CS-*g*-PA are suitable for copper removal.

For the removal of Pb (II) different adsorbents from biomass have been reported, such as: (a) wheat (*q_m_* = 87.0 mg·g^−1^; [60]); (b) rice husk ash (*q_m_* = 91.74 mg·g^−1^; [61]); (c) banana peel (*q_m_* = 2.18 mg·g^−1^; [62]); (d) microbial biomass from bacteria, such as *Pseudomonas putida* (*q_m_* = 270.4 mg·g^−1^; [63]); (e) *Streptomyces rimosus*, (*q_m_* = 135 mg·g^−1^; [64]); (f) fungi, such as *Sacharomyces cerevisiae* from beer production residues, (*q_m_* = 15.4 mg·g^−1^, [65]); (g) *Penicillium chrysogenum* (*q_m_* = 55 mg·g^−1^; [66]); (h) *Penicillium oxalycum*, from fermentation industry residues (*q_m_* = 47.4 mg·g^−1^; [67]); (i) algae such as *Sargassum* sp. (*q_m_* = 266 mg·g^−1^; [68]) and *Spirogyra* (*q_m_* = 140 mg·g^−1^; [69]). I our study, the CS had *q_m_* = 54 mg·g^−1^ and the CS-*g*-PA had *q_m_* = 80.6 mg·g^−1^, proving to be comparable or superior to many other systems used for this purpose.

All these results confirm the importance of the grafting reaction on the chitosan surface for improvement of the maximum adsorption capacity and the rate of the adsorption process, shaping as a potential useful adsorbent to removal Pb^2+^ and Cu^2+^ ions.

### 2.5. Adsorption Kinetics for Copper and Lead Ions

**Figure 5:**
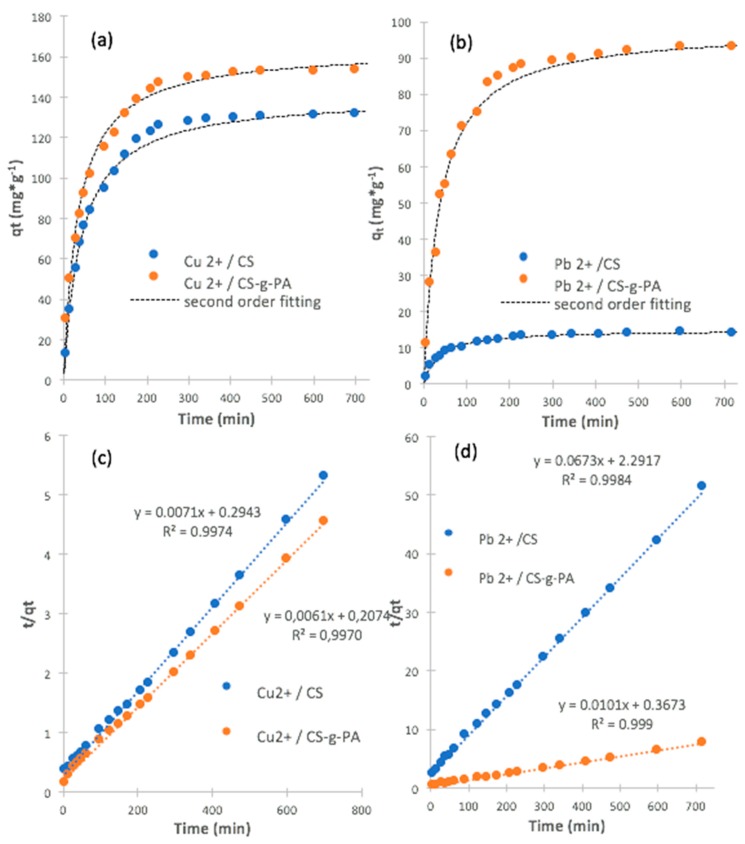
(**a**) Kinetic adsorption plots for Cu^2+^ on CS and CS-*g*-PA; (**b**) Kinetic adsorption plots for Pb^2+^ on CS and CS-*g*-PA; (**c**) linear fitting for Cu^2+^ on CS and CS-*g*-PA; (**d**) linear fitting for Pb^2+^ on CS and CS-*g*-PA; (initial concentration of Pb (II) and Cu (II) = 200 mg/L; pH = 4.0; adsorbent = 25 mg; volume of solution = 50 mL; temperature = 25 °C).

The study of adsorption kinetics is important to provide some information about the rate and mechanism of adsorption [70]. Figure 5 shows the variation in the adsorbed ions amount (*q_t_*) as a function of time. The rate of adsorption, for both ions, is high at initial times of adsorption. For both metals, most of the adsorption takes place within the first 100 min, using CS or CS-*g*-PA; however, after this time, we obtained near of 97% of the maximum adsorption. The sorption process is dependent on the sorbent characteristics such as the number of functional groups, porosity and amount of available sorption sites [71]. The pseudo-second order model is used to study the adsorption data; this model follows Equation (6) [72]:

(6) $$\;\frac{t}{q_{t}\;\;}=\frac{1}{k_{2}q_{e}^{2}}+\frac{t}{q_{e}}$$

where *q_t_* (mg/g) is the adsorption amount at time *t* (min), *k*_2_ (g/mg·min) is the rate constant of the pseudo-second-order kinetic adsorption. The values of *k*_2_ and *q_e_* are obtained from the intercept and regression coefficient of the experimental *t*/*q_t_* versus *t* data; Figure 5 shows pseudo-second model fitting and Table 3 lists kinetic parameters obtained from the fitting. Kinetic results revealed that the pseudo-second-order model has a suitable agreement with the experimental data. In this model, the rate-limiting step is the surface adsorption that involves chemisorption, where the removal from a solution is due to physicochemical interactions between the two phases [72,73]. Adsorption of Cu^2+^ on CS and CS-*g*-PA did not show appreciable differences in *k*_2_, however, *q_e_* was higher for CS-*g*-PA than CS. This result suggests that the grafting process increased chitosan surface affinity to Cu^2+^ ion. For Pb^2+^ removal, again *q_e_* was higher for CS-*g*-PA than CS. In this case, the *q_e_* was 6.6 times higher for CS-*g*-PA than CS. However, *k*_2_ was 7 times higher for CS than for CS-*g*-PA; it is well known that the chelation process of cationic ions on aminated surfaces is much lower rate than complexation with carboxylic group-bearing surfaces, which is mainly an electrostatic interaction process; despite *k_2_* reduces for CS-*g*-PA adsorbent, final amount of Pb^2+^ removal increased in a factor of 6, which indicates that the process is slower than CS, but CS-*g*-PA removes six times more Pb^2+^.

Lead and copper ions are known to exist in different forms in aqueous solutions at different pH values. At pH values below 6, Pb^2+^ is the major species, and with the increase of pH from 6 to 9, PbOH^+^ and Pb(OH)_2_ dominate [74]. Hence, by increasing pH over 6.0, the adsorption of lead and copper ions on chitosan may be enhanced, due to the attractive electrostatic interaction between chitosan and lead or lead hydroxide species. However, lead and copper attachment to chitosan can be reduced at pH lower than 6.0, as a consequence of repulsive electrostatic interactions. This phenomenon can be attributed at higher pH, to the presence of Pb^2+^ and Cu^2+^ hydroxide species (PbOH^+^, Pb(OH)_2_ and Cu(OH)_2_, respectively, which diffuse at a slower rate from the bulk of the solution to the surface of chitosan and requires a larger surface area for their attachment because of their larger size [49]. The carboxyl groups grafted from PA on CS-*g*-PA causes that adsorbent have greater negative zeta potentials, and therefore, CS-*g*-PA became more effective than CS for lead and copper ion adsorption during the equilibrium. However, the presence of –COOH groups cause that the diffusion of Pb^2+^ and PbOH^+^ to be slower than in the pristine chitosan, especially at higher pH (from 6.0), furthermore, also increase sites available for the ions attachment, a process that could be easier and faster for pristine chitosan and Pb^2+^; finally, at higher pH values lead and copper is removed from the solution by precipitation in form of Pb(OH)_2_ and Cu(OH)_2_.

## 3. Experimental

### 3.1. Materials

Chemical reagents were purchased from Aldrich (Palo Alto, CA, USA) and used without further purification unless otherwise stated. Chitosan was purchased from KOYO Chemical Co. (Osaka, Japan), M_w_ = 233 KDa, M_n_ = 105 KDa, PDI = 2.2, determined by GPC). Silicon substrates (silica wafers: SiO_2_ layers of 200 nm, doped with boron; face 100, p-type) were purchased from World KST Co. (Fukui, Japan), and were cut in the size convenient (typically 4 cm^2^) immersed in a mixture of sulfuric acid and hydrogen peroxide [piranha solution (3:1)] for a 10-min washing and then washed with deionized water (18.2 milliohms cm). 2-bromoisobutyryl bromide (2-BIB, 97%) was obtained from Alfa Aesar (Haverhill, MA, USA). CuBr, CuBr_2_, *N*,*N*,*N*′,*N*″,*N*″-pentamethyldiethylenetriamine (PMDETA), and triethylamine (99.5%) were purchased from Sigma-Aldrich (Palo Alto, CA, USA) and used as received. The standard solutions of Lead (II) and copper (II) of 1000 mg/L were purchased from Merck (Kenilworth, NJ, USA). Other chemicals were purchased from Sigma-Aldrich and used as received.

### 3.2. Synthesis

**Scheme 1:**
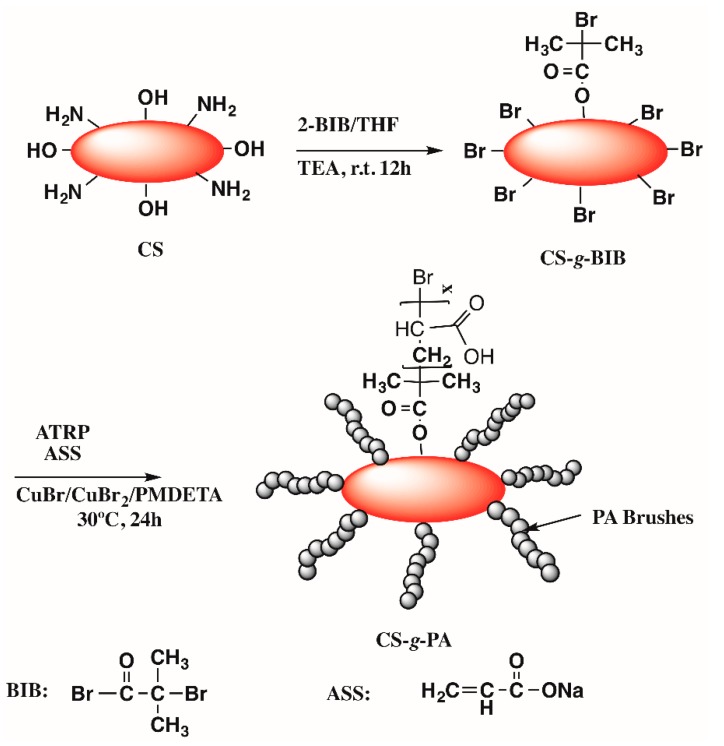
Schematic diagram for the synthesis of chitosan grafted polyacrylate from chitosan.

The ATRP initiator was grafted on the surface of chitosan (Scheme 1) using a modified reported procedure [75]: Five g of chitosan (deacetylation degree of 85%, medium molecular weight, Sigma-Aldrich) were added to a schlenk tube containing a solution of 25 mL of Tetrahydrofuran anhydrous (Sigma-Aldrich,) and 0.5 mL of triethylamine (99%, Aldrich). The tube was immersed in an ice bath and sealed using a septum. The content was gently stirred using a magnetic bar and purged with N_2_ during 30 min after what, 0.5 mL of 2-bromoisobutyryl bromide (2-BIB) (Sigma-Aldrich) was added under nitrogen dropwise placed in an ice bath. The reaction was removed from the ice bath and allowed to warm to room temperature and react overnight. Chitosan grafted BIB (CS-*g*-BIB) was filtered and washed extensively with THF, methanol and deionized water (DI), and allowed to dry in a vacuum oven at 40 °C for 48 h. 0.5 g of CS-*g*-BIB was carefully added to another schlenk tube with 21.51 mg (150 μmol) of CuBr (Sigma-Aldrich) and 3.35 mg (15 μmol) of CuBr_2_ (Sigma-Aldrich), sealed with a septum and flushed with N_2_ for 40 min. In a second Schlenk tube, 1.0 g (10.63 mmol) of sodium acrylate (Sigma-Aldrich) was added to a mixture of 111 μL (0.530 mmol) *of N*,*N*,*N*′,*N*″,*N*″-pentamethyldiethylenetriamine (PMDETA) and 17.5 mL of DI/methanol (1:1). The contents were gently stirred and degassed with nitrogen for 40 min and transferred using a cannula to the first tube. The polymerization proceeded for 24 h. CS-*g*-PA was washed with DI water in an ultrasonic bath, centrifuged and dried after water discard. The resultant chitosan was washed with large amounts of deionized water and followed was immersed in an acidic solution (0.1 M HCl) to remove any physically adsorbed reactant and to protonate the acrylic acid groups (ion exchange between Na^+^ to H^+^). Finally, the CS-*g*-PA obtained was dried in a vacuum oven at 40 °C for 48 h and stored in a desiccator for subsequent use [31].

### 3.3. Characterization

FT-IR spectra was analyzed with an Affinity FTIR spectrophotometer (Shimadzu, Columbia, MD, USA) equipped with a HgCdTe detector from 400 to 4000 cm^−1^, the percentage composition of chitosan was obtained with a FlashEA 1112 Series elemental analyzer (Thermo Electron, Waltham, MA, USA) Surface chitosan analysis, was performed using X-ray photoelectron spectroscopy, on a 5700 instrument (Physical Electronics, Chanhassen, MN, USA) using photoelectrons generated by the non-monochromatic Al KR irradiation (1486.6 eV). Photoelectrons were collected at a takeoff angle of 45° using a hemispherical analyzer operated in the fixed retard ratio mode with an energy resolution setting of 11.75 eV. The binding energy scale was calibrated prior to analysis using the Cu 2p3/2 and Ag 3d5/2 lines. Charge neutralization was ensured through bombardment of the irradiated area with an electron beam and the use of the non-monochromatic Al KR source. This placed the adventitious C 1s peak at a binding energy of 284.6 ± 0.2 eV.

### 3.4. Adsorption Assay

The study of initial pH effect on adsorption experiments were carried out by using 25 mg of the adsorbent (CS or CS-*g*-PA) in 50 mL of the initial metal solution at different pH (between 1.0–4.0) using an initial concentration of the metal of 200 ppm for 24 h at 25 °C under stirring. After the pH study, the adsorption experiments were carried out by using 25 mg of the adsorbent in 50 mL of the initial metal solution; for 24 h at 25 °C, at pH 4.0 under stirring; the solution concentrations were 100, 200, 300, 400 and 500 ppm; adsorption tests were carried out in duplicate. Metal concentration was determined by atomic absorption spectrophotometry for using calibration curves; Atomic absorption measurements were performed on a 200 Analyst system (Perkin Elmer, Waltham, MA, USA) using the flame method to determining the concentration of metal ion remaining in solution. NIST Lead and Copper standards (lead nitrate, Pb(NO_3_)_2_ and copper(II) sulfate, CuSO_4_ analytical grade were used to prepare calibration curves, with 1000 mg/L as stock solution and dilutions with concentrations of 1.0037, 1.9975, 3.0012, 3.995 and 4.9987 ppm, for copper and 1.000, 2.000, 3.000, 4.000 and 5.000 ppm for lead, with hollow cathode lamps at 324 nm for copper and 217 nm for lead determination. Finally, the studies of metal adsorption kinetics were carried out using at pH 4.0 for using 200 ppm as the initial concentration of the metal ion at a temperature of 25 °C and 25 mg of adsorbent under stirring.

## 4. Conclusions

In this work, we studied the implementation of chitosan grafted-polyacrylic acid as an absorbent to remove lead and copper from water. The kinetic experiments showed that CS-*g*-PA system had higher adsorption capacity for both metal ions and data were described by the pseudo-second-order kinetic model according to the *R* values. The maximum adsorption capacities obtained from the kinetic model were close to those from the Langmuir fitting model, except for lead CS adsorption, confirming chemical interactions between adsorbate and adsorbent species. We determined that a pH of 4.0 was the best pH for all the experiments according to the results. For the isotherm experiments, the Langmuir model was the best model describing the adsorption equilibrium process according to the linear regression and ARE values. The maximum adsorption capacity for Pb^2+^ ions was 98 mg·g^−1^ and for Cu^2+^ was 164 mg·g^−1^ over CS-*g*-PA. Finally, the results of this study confirm that chitosan-grafted polyacrylic acid can be used as a potential adsorbent to remove Pb^2+^ and Cu^2+^ ions from wastewaters, with reasonable amounts of adsorbent and consuming short periods of time.

## Figures and Tables

**Table 1:** Some methodologies for removal of Pb^2+^ and Cu^2+^ from water.

Ions	Method	Operation Condition	Removal Efficiency	Advantages/Disadvantages
Cu^2+^, Pb^2+^, Zn^2+^	Chemical precipitation	Cu^2+^ = 0.018 mM, Pb^2+^ = 2.3 mM, Zn^2+^ = 1.34 mM; precipitant, H_2_S; pH = 3.0	Cu^2+^ (100%), Pb^2+^ = (92%), Zn^2+^ = (94%)	Most widely used process in industry; it is relatively simple and inexpensive/it generates large volumes of relatively low density sludge; sulfide precipitants can result in the evolution of H_2_S [10,11].
Cu^2+^, Cr^3+^, Pb^2+^, Zn^2+^		[metal] = 100 mg·L^−1^ precipitant, CaO; pH = 3.0	>99%	
Cu^2+^	Ion exchange	Cu^2+^ = 100 mg·L^−1^; Resin-supported polyethyleneimine; pH = 5.0	>99%	High treatment capacity, high removal efficiency and fast kinetics/leaching during operation, highest costs for synthetic resins [9,11,12]
Pb^2+^; Cu^2+^		Cu^2+^ = 25 mg·L^−1^; Resin-THQSA; pH = 4.5	60–90%	
Cu^2^	membrane separation—Electrodyalisis	Cu^2+^ = 100 mg·L^−1^; membrane: packed beds of graphite powder; flow 1.29 × 10^−4^ Ls^−1^; current density 2 mAcm^2^; pH = 3.0	>99%	It has high efficiency, it requires little space, it is not selective and is easy to operate/it generates a large amount of metal-containing sludge [13].
Pb^2+^	Adsorption	Pb^2+^ = 50 mg·L^−1^; adsorbent: active carbon; pH = 6.0	>85%	Effective and economic method; it is flexible in design and operation and besides it can produce high-quality treated effluent; easy metal recovery [14,15,16]
Pb^2+^		Pb^2+^ = 50 mg·L^−1^; adsorbent: chitosan; pH = 4.5	>60%	
Cu^2+^		Pb^2+^ = 10 mg·L^−1^; adsorbent: chitosan; pH = 4.5	>80%	

**Table 2:** Theoretical Isothermal fitting experimental results.

Isothermal Model	Adsorbent	Metal Ion	Parameter (Unit)	Value
Langmuir	CS	Cu^2+^	*q_m_* (mg·g^−1^)	133
			*k_L_* (L·g^−1^)	29.8
			^1^ R^2^	0.998
			ARE	3.30%
		Pb^2+^	*q_m_* (mg·g^−1^)	54
			K (L·g^−1^)	42.6
			R^2^	0.998
			ARE	4.50%
	CS-*g*-TA	Cu^2+^	*q_m_* (mg·g^−1^)	136
			K(L·g^−1^)	32.3
			R^2^	0.998
			ARE	4.20%
		Pb^2+^	*q_m_* (mg·g^−1^)	80.6
			K (L/g)	39.6
			R^2^	0.997
			ARE	3.50%

^1^ fitting coefficient.

**Table 3:** Model kinetic parameters for removal Pb^2+^ and Cu^2+^ by CS and CS-*g*-PA.

Adsorbent	Metal Ion	^1^*k*_2_ (g·mg^−1^·min^−1^) × 10^−4^	*q_e_* (mg·g^−1^)	R^2^
CS	Cu^2+^	1.7	140	0.997
	Pb^2+^	19.8	14.8	0.998
CS-*g*-PA	Cu^2+^	1.8	164	0.997
	Pb^2+^	2.8	98.0	0.999

^1^ rate constant of the pseudo-second-order kinetic adsorption.

## Supplementary Materials

The following are available online, Table S1: Theoretical Isothermal fitting experimental results.
