# Supplementary material for: Equilibrium and Kinetic Study of Lead and Copper Ion Adsorption on Chitosan-Grafted-Polyacrylic Acid Synthesized by Surface Initiated Atomic Transfer Polymerization
Source: Molecules. 2018 Sep 1;23(9):2218. doi: 10.3390/molecules23092218 (PMC6225132; doi:10.3390/molecules23092218)
Supplement: Supplementary file 1 [file molecules-23-02218-s001.pdf]

Table S1. Theoretical Isothermal fitting experimental results.

Isothermal Model	Adsorbent	Metal ion	Parameter (unit)	Value
Freundlich	CS	Cu^{2+}	n	2.5
			k	7.1
			R^2	0.867
			ARE	13.9
		Pb^{2+}	n	2.4
			k	4.1
			R^2	0.851
			ARE	13.9
	CS-g-TA	Cu^{2+}	n	2.4
			k	7.0
			R^2	0.891
			ARE	15.2
		Pb^{2+}	n	2.4
			k	5.2
			R^2	0.88
			ARE	12.6
Temkin	CS	Cu^{2+}	A	2.3
			B	25
			R^2	0.978
			ARE	3.7%
		Pb^{2+}	A	2.5
			B	9.7
			R^2	0.985
			ARE	4-0%
	CS-g-TA	Cu^{2+}	A	2.0
			B	26
			R^2	0.979
			ARE	5.1%
		Pb^{2+}	A	2.6
			B	15.2
			R^2	0.990
			ARE	4.7%

[1] fitting coefficient.
